# The Fc Receptor-Like 3 Polymorphisms (rs7528684, rs945635, rs3761959 and rs2282284) and The Risk of Neuromyelitis Optica in A Chinese Population

**DOI:** 10.1097/MD.0000000000001320

**Published:** 2015-09-25

**Authors:** Wenjing Lan, Shaokuan Fang, Huimao Zhang, Dan Tong Jianmeng Wang, Jiang Wu

**Affiliations:** From the Department of Radiology, the First Hospital of Jilin University, Changchun, Jilin Province 310000, China (WL, HZ, DT); Department of Neurology, the First Hospital of Jilin University, Changchun, Jilin Province 310000, China (SF, JW); and Department of Geriatrics, the First Hospital of Jilin University, Changchun, Jilin Province 310000, China (JW).

## Abstract

Neuromyelitis optica (NMO) appears to be a severe inflammatory demyelinating disease occurring in the central nervous system. Furthermore, the Fc receptor-like 3 (*FCRL3*) gene was previously found to be susceptible for a certain inflammatory demyelinating diseases (such as multiple sclerosis). The present study, therefore, was aimed to explore the possible association of *FCRL3* gene polymorphisms with susceptibility to NMO in a Chinese Han population.

Seven single nucleotide polymorphisms (SNPs) of *FCRL3* were, respectively, genotyped in 132 NMO patients and 264 healthy controls via PCR assay. Moreover, the *t*-test and the chi-square test were used to estimate the association between genetic mutations of *FCRL3* and the risk of NMO with Statistical Analysis System (SAS) software (Version 9.0).

It was demonstrated that FCRL3_3, 5, 6 and 8, SNPs were remarkably associated with susceptibility to NMO in both allelic [OR = 1.50 (95% CI: 1.11–2.03, *P* = 0.008), OR = 1.44 (1.07–1.94, *P* = 0.015), OR = 1.45 (1.08–1.95, *P* = 0.014), and OR = 2.01 (1.13–3.60, *P* = 0.016)] and homozygous models [OR = 2.19 (95% CI: 1.19–3.99, *P* = 0.010), OR = 2.09 (1.15–3.80, *P* = 0.014), OR = 2.04 (1.13–3.67, *P* = 0.016), and OR = 5.33 (1.02–27.9, *P* = 0.027)]. However, the other 4 SNPs, FCRL3_4, FCRL3_7, FCRL3_9, did not show the significant associations with NMO.

Conclusions in the present study could be drawn that 4 SNPs in *FCRL3* (FCRL3_3∗C, 5∗C, 6∗A, 8∗G) might account for increased risk of NMO in a Chinese-Han population. Nevertheless, further cohort studies are in demand to validate the association in the future.

## INTRODUCTION

Neuromyelitis optica (NMO), also called Devic's disease, seems to be a severe inflammatory demyelinating disease developing in the central nervous system that is featured by prior invasion of the optic nerves and spinal cords.^1,2^ NMO is predominantly diagnosed in females, and it also occurs in children and elderly people.^2–7^ Moreover, NMO could be found in plenty of East Asians and other non-white populations.^2,3^ In fact, during a long time, NMO was confused by investigators with multiple sclerosis due the facts that prognosis then was poor^1^ and that both NMO and multiple sclerosis belonged to the inflammatory demyelinating disease family. At present, the clinical and laboratory features of NMO have been recognized by several independent studies.^2,3,8^ Specifically, the discovery of a specific NMO-immunoglobulin G (NMO-IgG) autoantibody against the aquaporin-4 water channel in NMO patients was confirmed by different experiments, and magnetic resonance imaging findings of NMO were in accordance with the presence of longitudinally extensive myelitis.^9,10^ More than that, the studies published before have suggested that both genetic and environmental factors could affect the inflammatory demyelinating disease. Additionally, the familial occurrence of NMO cases is more common than expected, suggesting a complex genetic susceptibility of NMO.^11^

As is reported, the Fc receptor-like (*FCRLs*) gene family encodes the members of the immunoglobulin receptor superfamily and they are concentrated on the long arm of chromosome 1, which contains 6 subtype genes (*FCRL1*, *FCRL2*, *FCRL3*, *FCRL4*, *FCRL5*, *FCRL6*). It was also investigated that the gene family could regulate the *FCRL* expression by altering the binding affinity of nuclear factor-κB.^12^ Among the 6 subtype genes, *FCRL3* and *FCRL5* have been identified to be correlated with several autoimmune diseases, such as autoimmune thyroid disease, rheumatoid arthritis, systemic lupus erythematosus, and so on.^13–15^ In particular, multiple sclerosis, an inflammatory demyelinating disease, has been demonstrated to be associated with *FCRL3* mutation in Spanish and Japanese studies.^12,16^ However, although NMO has also been considered to be an inflammatory demyelinating disease and an autoimmune disease, there were no studies that were concentrated on the association between the risk of NMO and polymorphisms in *FCRL3*. Moreover, the association of the single nucleotide polymorphisms (SNPs) in *FCRL3* with Graves’ disease was also replicated in a Japanese study.^12^ The polymorphisms in *FCRL5* might also impose secondary effects on the *FCRL3* gene,^17^ cumulatively influencing the development of Graves’ disease. Considering the above complicated effects of polymorphisms in *FCRL3* on the occurrence of certain diseases of great significance, a specific study was required to estimate the relationship between genetic mutations in *FCRL3* and the risk of NMO. The aim of the present study, therefore, was to investigate the association between SNPs in *FCRL3* and susceptibility to NMO in a Chinese Han population.

## MATERIALS AND METHODS

### Ethic Statement

All patients have signed written informed-consent forms before participating in this study, and the present study was approved by the First Hospital of Jilin University.

### Participants

A retrospective case-control study was performed to investigate the association between SNPs of *FCRL3* and the risk of NMO. The participants were made up of 132 NMO patients (male/female = 62/70) and 264 healthy controls (male/female = 128/136). The NMO patients were recruited between April 2012 and March 2014 and they satisfied the 2006 criteria for diagnosis of NMO.^32^ All of the involved patients were ascertained by clinical neurologists and were then confirmed with clinical laboratory NMO-IgG seropositive tests. The control subjects were diagnosed as healthy individuals without history of any cancer through routinely clinical and physical examinations in the outpatient departments during the same period. Apart from that, the age and sex ratio of healthy controls were matched with NMO patients. Furthermore, there was no genetic association between NMO patients and control subjects. The relevant clinical data were available from the participants’ medical records.

### SNP Selection

The International HapMap Project database (HapMap Data Rel 24/phaseII Nov08, on NCBI B36 assembly, dbSNP b126) contains the genotyped data from Chinese Han individuals without genetic associations. Based on the above database, the target SNPs were selected with usage of HaploView software (version 4.2) if the following criteria were all satisfied: (1) minor allele frequency (MAF) was >0.05, (2) *P* value of Hardy–Weinberg equilibrium (HWE) was >0.1, (3) *r*^2^ was greater than 0.8. Besides, several previously reported SNPs were also under consideration.

### SNP Genotyping

First, 10 ml venous blood was collected from each patient with the ethylene diamine tetraacetic acid tube. Then, the Blood DNA Extraction kits II (Beijing Bioteke Co. Ltd) was used to extract the genomic DNA from venous blood. The polymerase chain reaction (PCR) assay was subsequently executed with TaqMan Mior Groove Binder (MGB) chemistry (Applied Biosystems, Foster City, CA) to identify the target SNPs. To be specific, DNA (10 ng), TaqMan Master Mix (2.5 μl), assay mixture (0.065 μl), and distilled, DNase-free water (2.435 μl) were mixed together for each PCR assay. After that, the SNPs were amplified following different protocols (Table 1). Finally, the PCR products were sequenced directly with a DNA sequencing Kit and the Big Dye Terminator on an automated ABI PRISM 3100 DNA sequence detection system (Applied Biosystem, Forster City, CA). Furthermore, the genotyping accuracy of the above results was confirmed with random samples detected by TaqMan.

**TABLE 1:**
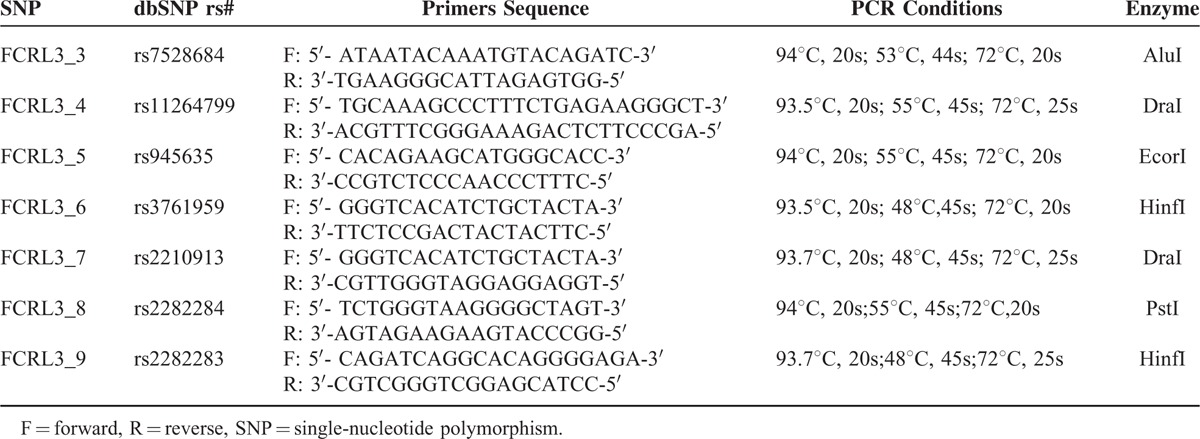
Primers of *FCRL3* Gene Polymorphisms for PCR Amplification

### Statistical Analysis

*P* value of HWE was calculated with HaploView software (Version 4.2) to measure the genotyping distributions for the cases and healthy controls. The Haplotype frequencies and linkage disequilibrium were also calculated with the same software. Moreover, the chi-square test, coupled with the odds ratio (OR) and its 95% confidence interval (95%CI), was used to examine the association between selected SNPs and the risk of NMO in allelic, dominant, recessive, and homozygous models. Additionally, the differences between case and control groups in certain variables, such as age and sex, were identified using the *t*-test and Pearson's chi-square test. All of the above statistical tests were performed with Statistical Analysis System (SAS) software (Version 9.0).

## RESULTS

### Characteristics of SNPs

**FIGURE 1:**
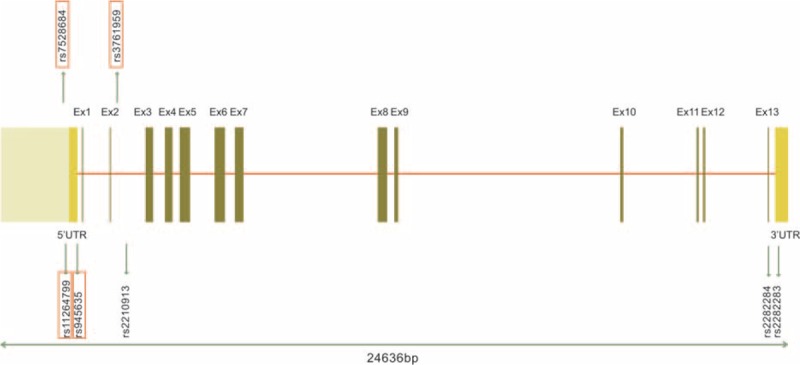
Genetic location of the 7 target SNPs in the *FCRL3* gene.

A total of 7 SNPs (rs7528684, rs11264799, rs945635, rs3761959, rs2210913, rs2282284, rs2282283) of *FCRL3* gene were selected for this study in accordance with HWE. The detailed information about each polymorphism, such as primer sequence, PCR assay condition and enzyme, were displayed in Table 1. Moreover, Figure 1 shows the relative positions of the 7 selected SNPs in the *FCRL3* gene. Specifically, rs7528684 and rs11264799 are approximately near the promoter region (5′), whereas rs945635 is exactly in the promoter region. Besides, rs2282283 is situated in the terminator region and rs2282284 is located in the exon 13 region. The remaining 2 SNPs lie in the intron 2 region.

### Characteristics of Participants

The demographic and clinical characteristics of NMO patients and healthy groups are shown in Table 2. The sex distribution (male/female) was 62/70 in NMO patients group and 128/136 in the healthy group. No significant difference between cases and control subjects was observed in mean age (*P* = 0.196) and sex ratio (*P* = 0.776). The average onset age (± SD) and duration of NMO (± SD) were 40.1 (± 11.9) and 6.5 (± 5.3), respectively. In addition, more data about clinical variables were collected for NMO patients’ group, such as annual relapse rate, positive AQP4-Ab, visual activity, abnormal brain MRI at last test, and so on.

**TABLE 2:**
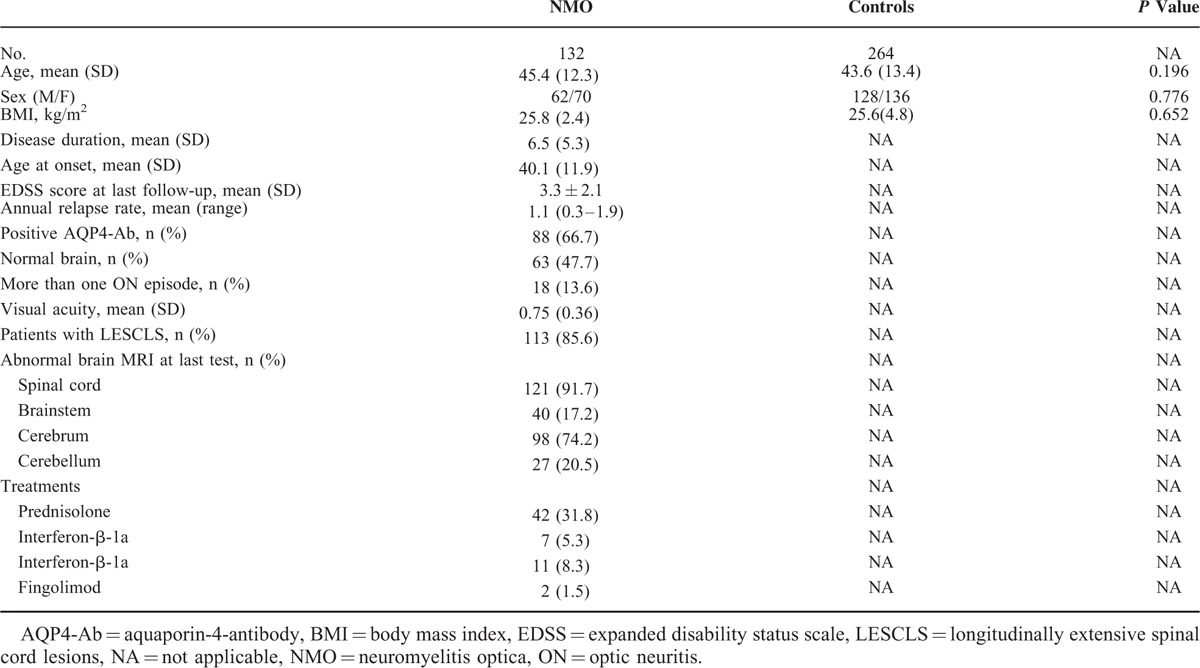
Demographic and Clinical Characteristics and Treatment in NMO Patients

### Distribution of Genotype Frequency and the Risk of NMO

**FIGURE 2:**
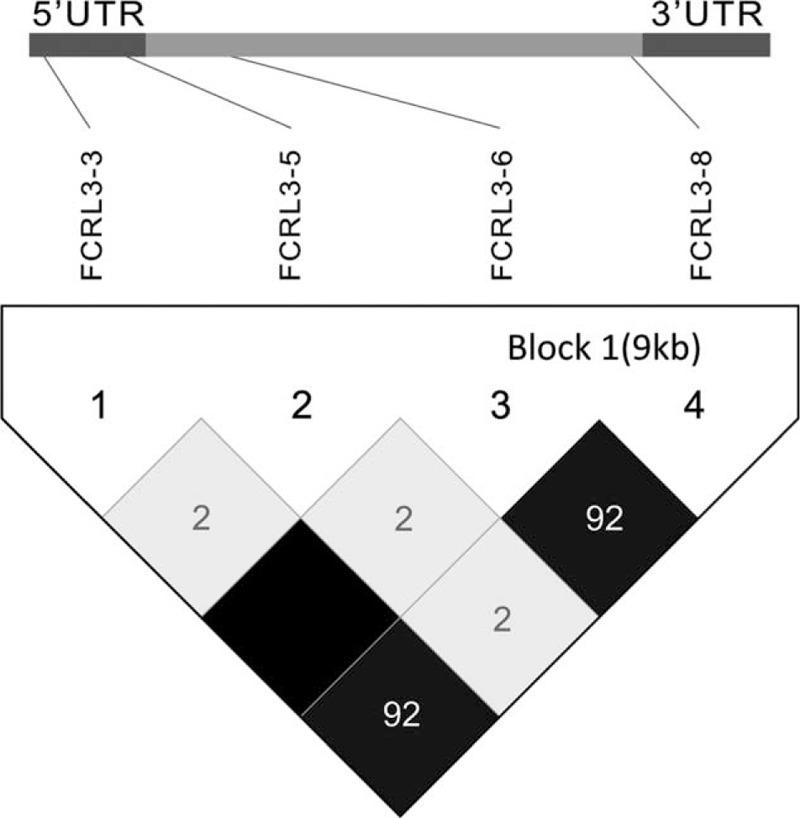
Linkage disequilibrium (LD) of the 4 SNPs in the *FCRL3* gene.

The case-control analysis demonstrated that rs7528684 (FCRL3_3), rs945635 (FCRL3_5), rs3761959 (FCRL3_6), and rs2282284 (FCRL3_8) showed significant associations with risk of NMO, whereas other 3 SNPs were not. To be specific, the FCRL3_3∗C, FCRL3_5∗C, FCRL3_6∗A, FCRL3_8∗G allelic frequencies were significantly higher in the case group than those in the control group (OR = 1.50, 95% CI: 1.11–2.03, *P* = 0.008; OR = 1.44, 95% CI: 1.07–1.94, *P* = 0.015; OR = 1.45, 95% CI: 1.08–1.95, *P* = 0.014; OR = 2.01, 95% CI: 1.13–3.60, *P* = 0.016). Moreover, their recessive models (except rs3761959) and homozygous models also revealed the remarkable associations between the genetic variants and the risk of NMO. However, the dominant models or the allelic models failed to show any significant correlations between the rest 3 SNPs and the risk of NMO (Table 3). In addition, haplotype analysis showed that FCRL3_3∗C, FCRL3_6∗A, and FCRL3_8∗G were in a strong linkage disequilibrium (LD), except FCRL3_5 (Figure 2).

**TABLE 3:**
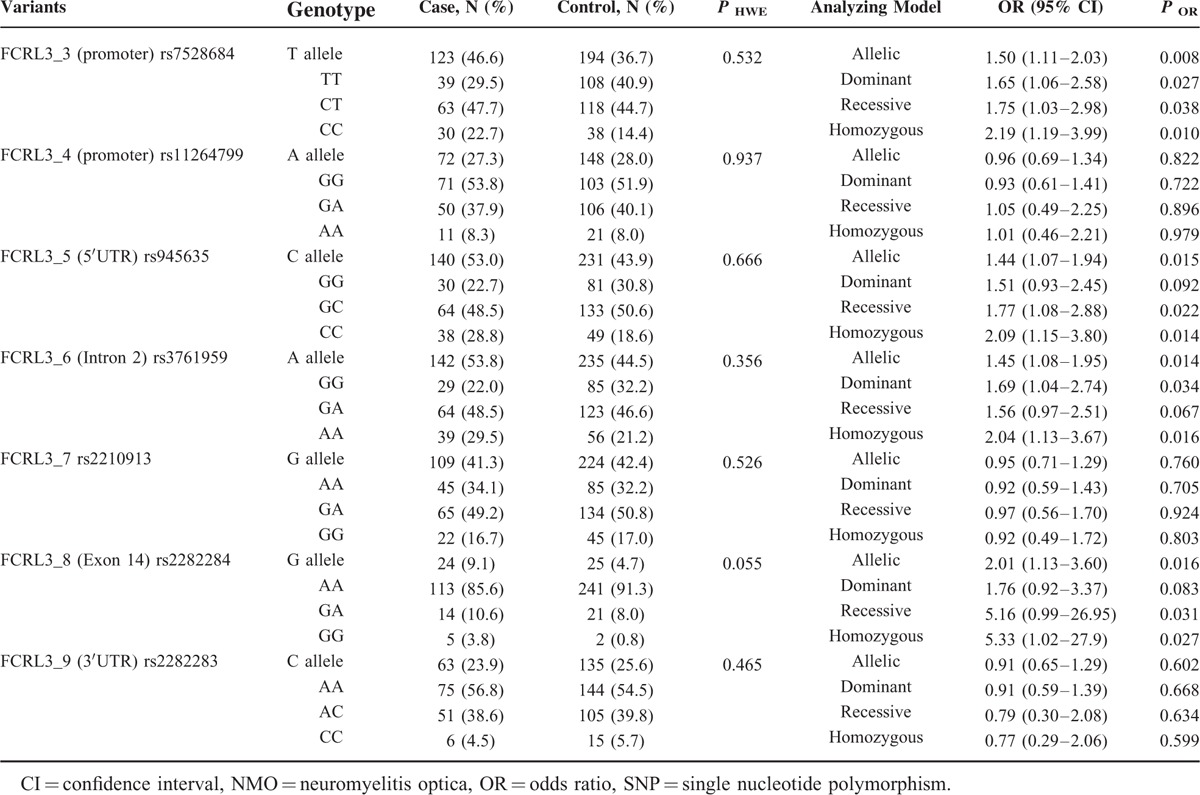
Allele and Genotype Distributions of *FCRL3* SNPs in NMO Patients and Controls

## DISCUSSION

The present study revealed an association between genetic mutations in *FCRL3* (rs7528684, rs945635, rs3761959, and rs2282284) and elevated risk of NMO in a Chinese Han population. The notable associations were confirmed with homozygous and allelic models as well.

As is demonstrated, *FCRL3* encoded a member of the immunoglobulin receptor superfamily, which could directly act against myelin-derived antigens.^16^ Furthermore, although the precise function of *FCRL3* remains to be unknown, the contained immunoreceptor-tyrosine inhibitory motifs (ITIMs) and immunoreceptor-tyrosine activation motifs (ITAMs) are deemed to be involved in the regulation of the immune system.^18^ More specifically, both ITIMs and ITAMs are in the cytoplasmic domain, indicating that this membranous receptor participates in transduction of the signal into the cell through its cytoplasmic tail.^19^ Based on the above basic features of *FCRL3*, plenty of studies have revealed the association of polymorphisms of *FCRL3* with susceptibility to several autoimmune disorders.^20–23^ Nevertheless, there has been no study reporting the association between polymorphisms of *FCRL3* and the risk of NMO in the Chinese population. Therefore, the present study, up to date, was the first investigation focused on the association.

This study demonstrated that the 4 polymorphisms (rs7528684, rs945635, rs3761959, and rs2282284) of *FCRL3* could account for an elevated risk of NMO. The FCRL3_3 (rs7528684) is common in previous studies, which have reported that plenty of autoimmune disorders were associated with this polymorphism.^24–26^ More specifically, the association of *FCRL3_3*C allele with rheumatoid arthritis has been investigated in both Japanese and Canadian populations.^13,20,21,27^ Although this association failed to be replicated in independent studies for certain Europeans, who reside in North America, UK, and Spain, a meta-analysis further confirmed the susceptibility of rheumatoid arthritis in Asians, rather than Europeans.^21,28^ In fact, the frequency of putative disease causal allele of the FCRL3_3 is similar in Caucasians (40%) and Asians (35%).^19^ Therefore, the dissimilar associations of Caucasians and Asians with this autoimmune disorder could not be explained by the ethnic distinctions alone. Instead, the unique environmental or the geographical conditions could trigger genetic variations that are relevant to the susceptibility to rheumatoid arthritis. Apart from rheumatoid arthritis, Graves’ disease and Behcet's disease were also observed to be significantly associated with this polymorphism in the *FCRL3* gene.^23,29^

Furthermore, a Japanese study^12^ and a couple of independent Spanish studies^16,30^ have identified the association of FCRL3_3 polymorphisms with susceptibility to multiple sclerosis among Asians and Caucasians, respectively. However, opposite conclusions regarding the association were suggested. In the present study, the SNPs that have been considered to be associated with the multiple sclerosis^16,30^ were assumed to be significantly correlated with risk of NMO as well. Moreover, there exist no notable associations of FCRL3_4 with susceptibility to both multiple sclerosis in the Spanish study^30^ and NMO in the present study. The above phenomena could be explained by the fact that the etiology of multiple sclerosis and that of NMO still possess some similar points despite some dissimilarities^31,32^ to some extent, for example, the presence of multiple sclerosis and NMO are both partly due to the invasion of the immune system through the central nervous system under misdirection. Nonetheless, rs945635 (FCRL3_5), rs3761959 (FCRL3_6), and rs2282284 (FCRL3_8) that were previously not indicated to be associated with risk of multiple sclerosis or NMO displayed a remarkable association with susceptibility to NMO in the present study. Among the 3 SNPs, the polymorphisms of FCRL3_6 and FCRL3_8, which are situated in the coding region, could easily cause changes in amino acids, indicating their positive effects on the risk of NMO in the Chinese populations. Whereas the genetic mutations of *FCRL3* had been rarely studied in the association with risk of NMO, there were several studies suggesting the significant associations of *FCRL3* polymorphisms with multiple sclerosis. After comprehensively comparing this study results with previous studies, conclusions could be drawn that the polymorphisms in *FCRL3* might have similar associations with both multiple sclerosis and NMO, even though the sample size and ethnicities in diverse studies regarding multiple sclerosis and NMO were different.

Although *FCRL3* polymorphisms are considered to be potentially associated with risk of NMO in the present study, some limitations still exist and they need to be addressed in the future. First, the limited number of patients and controls enrolled in the study could have interfered with the statistical power, for example, the *P* value of HWE for the selected polymorphisms was reduced. Second, the study subjects were not fully representative since that the study was hospital-based and that the gender ratio in the case group was not consistent with the standard ratio, which was concluded from a review study.^1^ To make up for this disparity, subgroup analysis on the basis of gender could help us to understand whether the disease distribution differs between males and females. Last, the study in terms of additional relevant polymorphisms would be in urgent demand to explore the actual effects of *FCRL3* genetic mutations on NMO.

In conclusion, the association analyses between *FCRL3* polymorphisms and susceptibility to NMO have been conducted in the present study. The results demonstrated that 4 SNPs (rs7528684, rs945635, rs3761959, and rs2282284) could significantly elevate the risk of NMO. Even though the functional background of the above 4 SNPs has been partly concluded based on previously published studies, certain specific mechanisms still remain vague. The present study would play a valuable part in investigating the etiology of NMO among Asians through identifying the significant association between *FCRL3* polymorphisms and the risk of NMO in a Chinese Han population. Nevertheless, further investigations are required to confirm the functional role of *FCRL3* polymorphisms on NMO and more guidance for treatments of NMO would thus be provided.
